# Effect of fish oils containing different amounts of EPA, DHA, and antioxidants on plasma and brain fatty acids and brain nitric oxide synthase activity in rats

**DOI:** 10.3109/03009730903268958

**Published:** 2009-12-08

**Authors:** Karin Engström, Ann-Sofie Saldeen, Baichun Yang, Jawahar L. Mehta, Tom Saldeen

**Affiliations:** ^1^Department of Surgical Sciences, University of Uppsala, UppsalaSweden; ^2^Department of Pediatrics, Rigshospitalet, Bispebjerg Hospital, CopenhagenDenmark; ^3^Division of Cardiovascular Medicine, University of Arkansas for Medical Sciences, Little Rock, ARUSA

**Keywords:** Antioxidants, brain, DHA, EPA, fish oil, lipid peroxidation, nitric oxide synthase

## Abstract

**Background:**

The interest in n-3 polyunsaturated fatty acids (PUFAs) has expanded significantly in the last few years, due to their many positive effects described. Consequently, the interest in fish oil supplementation has also increased, and many different types of fish oil supplements can be found on the market. Also, it is well known that these types of fatty acids are very easily oxidized, and that stability among supplements varies greatly.

**Aims of the study:**

In this pilot study we investigated the effects of two different types of natural fish oils containing different amounts of the n-3 PUFAs eicosapentaenoic acid (EPA) and docosahexaenoic acid (DHA) and antioxidants on plasma and brain fatty acids, blood lipids, vitamin E, and *in vivo* lipid peroxidation, as well as brain nitric oxide synthase (NOS) activity, an enzyme which has been shown to be important for memory and learning ability.

**Methods:**

Sprague-Dawley rats were divided into four groups and fed regular rat chow pellets enriched with 5% (w/w) of butter (control group), a natural fish oil (17.4% EPA and 11.7% DHA, referred to as EPA-rich), and a natural fish oil rich in DHA (7.7% EPA and 28.0% DHA, referred to as DHA-rich). Both of the fish oils were stabilized by a commercial antioxidant protection system (Pufanox^®^) at production. The fourth group received the same DHA-rich oil, but without Pufanox^®^ stabilization (referred to as unstable). As an index of stability of the oils, their peroxide values were repeatedly measured during 9 weeks*.* The dietary treatments continued until sacrifice, after 10 days.

**Results:**

Stability of the oils varied greatly. It took the two stabilized oils 9 weeks to reach the same peroxide value as the unstable oil reached after only a few days. Both the stabilized EPA- and DHA-rich diets lowered the triacylglycerols and total cholesterol compared to control (-45%, *P* < 0.05 and -54%, *P* < 0.001; -31%, *P* < 0.05 and -25%, *P* < 0.01) and so did the unstable oil, but less efficiently. Only the unstable oil increased *in vivo* lipid peroxidation significantly compared to control (+40%, *P* < 0.001). Most of the fatty acids in the plasma phospholipids were significantly affected by both the EPA- and DHA-rich diets compared to control, reflecting their specific fatty acid pattern. The unstable oil diet resulted in smaller changes, especially in n-3 PUFAs. In the brain phospholipids the changes were less pronounced, and only the diet enriched with the stabilized DHA-rich oil resulted in a significantly greater incorporation of DHA (+13%, *P* < 0.01), as well as total n-3 PUFAs (+13%, *P* < 0.01) compared to control. Only the stabilized DHA-rich oil increased the brain NOS activity (+33%, *P* < 0.01).

**Conclusions:**

Both the EPA- and DHA-rich diets affected the blood lipids in a similarly positive manner, and they both had a large impact on plasma phospholipid fatty acids. It was only the unstable oil that increased *in vivo* lipid peroxidation. However, the intake of DHA was more important than that of EPA for brain phospholipid DHA enrichment and brain NOS activity, and the stability of the fish oil was also important for these effects.

## Introduction

In the last few years, the interest in n-3 polyunsaturated fatty acids (PUFAs) has expanded significantly due to the many positive effects described. As a consequence, the interest in fish oil supplementation has also increased considerably, and many studies have demonstrated the benefits to different conditions, including cardiovascular (1,2), neurological, and psychiatric disorders (3). Many different types of fish oil supplements can be found on the market, but studies comparing them are rare. In this pilot study we aimed at investigating two different types of fish oils containing high amounts of either the n-3 PUFA eicosapentaenoic acid (EPA) or docosahexaenoic acid (DHA). They were both of natural origin and thus not chemically modified to contain the pure fatty acid. Besides cardiovascular markers, we also studied the brain for fatty acids and nitric oxide synthase (NOS) activity. This enzyme has been shown to be of critical importance for memory and learning ability (4–9).

It is also well known that these highly unsaturated fatty acids are highly susceptible to oxidation, thereby making the production of a stable fish oil very difficult (10). Consequently, the stability among supplements varies greatly (11). Several studies have shown that intake of an unstable oil can induce lipid peroxidation which may affect the results negatively and may even produce adverse effects (12–14). Thus, in order to elucidate the importance of *in vitro* stability of the fish oil for *in vivo* effects, we included one of the oils (the DHA-rich) which had been treated differently at production as it was not stabilized by a sufficient amount of antioxidants and consequently was shown to be unstable.

## Materials and methods

### Animals and diets

Male pathogen-free Sprague-Dawley rats (*n* = 24) weighing 250-350 g were fed standard pellet diets containing 57% carbohydrates, 17% protein, 1.4% fat, and 74 mg vitamin E/kg. The standard pellet diets were enriched with 5% (w/w) of butter (control, n = 6) or with 5% (w/w) of three different fish oil preparations as follows: One group of rats (*n* = 6) received a natural fish oil rich in EPA (referred to as EPA-rich oil). Another group of rats (*n* = 6) received a natural fish oil rich in DHA (referred to as DHA-rich oil). These oils were stabilized with an antioxidant protection system (Pufanox^®^) in exactly the same way. The last group (*n* = 6) received the same DHA-rich oil as the third group did, but without the antioxidant protection system. The four different groups are referred to as control, stabilized EPA-rich oil, stabilized DHA-rich oil, and unstable DHA-rich oil. All diets were stored in sealed containers under nitrogen at 4°C in the dark and supplied to the animals once daily. After 10 days the rats were weighed, anaesthetized with pentobarbital sodium (60 mg/kg body weight) given intraperitoneally, and then killed by exsanguination. Plasma was prepared immediately by centrifugation of the EDTA-rich blood for 15 min at 500 *g* at 4°C, and then stored at -70°C. The brain was excised, weighed, divided into smaller pieces, and stored at -70°C.

### Fish oil and antioxidant compositions

Both fish oils were natural and had not been chemically modified. The EPA-rich oil (ESKIMO-3, Cardinova, Uppsala, Sweden) was obtained from small sardines living in cold deep water, and it represents a DHA:EPA ratio normally found in a typical, not chemically altered, natural fish oil. The DHA-rich oil (EPAX 0525TG, Pronova, Oslo, Norway) was obtained from the eyes of tuna fish. The fatty acid compositions, as assayed by gas-liquid chromatography, of the different fish oils and butter are shown in Table I. The supplements contained approximately equal amounts of monounsaturated and n-6 fatty acids. The saturated fat in the control (butter) diet was replaced by n-3 PUFAs in the fish oil diets. The cholesterol concentration was less than 3 mg/g both in the butter and fish oil supplements. The EPA-rich oil (ESKIMO-3) was stabilized using a commercial natural antioxidant mixture (Pufanox^®^, Cardinova, Uppsala, Sweden) which includes tocopherols and vitamin C among other components. The DHA-rich oil was stabilized in exactly the same way (Cardinova, Uppsala, Sweden). The final *d*-α-tocopherol concentration in the stabilized oils was 3.5 IU/g. The unstabilized oil contained the original concentration of 0.75 IU *d*-α-tocopherol/g. The butter contained 0.03 IU *d*-α-tocopherol/g. As an index of the stability of the oils, the peroxide values were repeatedly measured during a 2-month period. The oils were stored in the dark, in open beakers, and at room temperature. The peroxide values were measured according to American Oil Chemists' Society (AOCS) Official Method Ca 18-90.

**Table I.** Fatty acid composition of the supplements.^a^

	Supplement		
Fatty acid^b^	Butter	EPA-rich oil	DHA-rich oil
4:0-12:0	14.6	-	-
14:0	11.1	8.3	4.2
16:0	27.4	15.2	19.2
18:0	10.5	2.6	4.3
Total saturated	63.6	26.9	28.6
16:1(n-7)	3.1	9.1	7.0
18:1(n-7+n-9)	23.6	11.2	14.5
20:1(n-9)	-	1.1	1.2
Total monounsaturated	26.7	22.6	23.1
18:2(n-6)	2.6	1.2	1.6
16:3(n-4)	-	1.8	1.0
16:4(n-3)	-	3.2	-
18:4(n-3)	-	3.4	1.2
20:4(n-6) AA^c^	-	1.0	2.2
22:4(n-3)	-	-	1.8
20:5(n-3) EPA	-	17.4	7.7
22:5(n-3)	-	2.1	1.0
22:6(n-3) DHA	-	11.7	28.0
Total (n-6) PUFAs	2.6	2.3	3.8
Total (n-3) PUFAs	-	40.2	40.9
Ratio EPA:total (n-3)	-	0.4	0.2
Ratio DHA:total (n-3)	-	0.3	0.7

^a^In g/100 g total fatty acids. The standard pellet diet (1.4% fat, w/w) was enriched with 5% (w/w) butter (control) or fish oils. The fatty acid composition of the butter was reported by the manufacturer. ^b^Only fatty acids ≥1.0 g/100 g total fatty acids are shown. ^c^Arachidonic acid.

### Analyses of blood samples

Blood lipoproteins (total cholesterol and triacylglycerols) were measured immediately after blood sampling, using enzymatic methods.

For the measurement of *in vivo* lipid peroxidation, lipoperoxides (generated *in vivo* as a result of oxygen free-radical-induced lipid peroxidation) were hydrolysed, and the products including malondialdehyde (MDA) were reacted with thiobarbituric acid (TBA). The resulting MDA-(TBA)_2_ was measured by reversed phase high-performance liquid chromatography (RP-HPLC). The method has been described previously (15); however, some modifications were made as described elsewhere (16). The results are expressed as μmol/L MDA equivalents.

For measurements of plasma α-tocopherol concentrations, a RP-HPLC system was used. The samples were treated according to Öhrvall *et al*. (17). For  detection an ultraviolet (UV), instead of fluorescence, detector was employed (AOCS Official Method Ce 8-89, 1990).

For measurement of fatty acids, lipids in plasma or brain homogenates (all brains were prepared by the same person and extracted from the same location of cerebral cortex) were extracted with chloroform/methanol and submitted to thin-layer chromatography (TLC), and the phospholipids were subsequently recovered after evaluation of the plate. After hydrolysis and transmethylation, the fatty acid methyl esters were separated by gas-liquid chromatography as previously described (18).

### Analyses of brain NOS

The NOS activity in brain homogenates was measured by monitoring the conversion of (^3^H)arginine to (^3^H)citrulline as described earlier (19). Briefly, the reaction mixture consisted of 100 μg of brain homogenate and 100 μL of (^3^H)arginine (100 nmol/L) to 1 ml of buffer containing 50 mmol/L Hepes (pH 7.4), 1 mmol/L NADPH, 1 mmol/L EDTA, 1.25 mmol/L CaCl_2_, 1 mmol/L dithiothreitol, and 10 μg/mL calmodulin. After incubation for 30 min at 37°C, the reaction was terminated, and 1 ml of the mixture was applied to Dowex AG50WX-8 (Na+ form, Dow Pharmaceuticals Co., Midland, MI) columns (Bio-Rad, Hercules, CA) and eluted with 2 ml of distilled water. ^3^H-L-citrulline was counted, and NOS activity was expressed as nmol/mg protein. Total protein content was quantified by bicinchoninic acid (BCA) protein assay kit (Pierce, Rockford, IL).

### Statistical methods

The SPSS 10.1 software package (SPSS, Inc., Chicago, Illinois, USA) was used for the statistical analyses. Multiple comparisons were made by one-way ANOVA followed by the *post hoc* least square (LSD) test. *P*-values <0.05 were adopted as significant. Data are presented as mean ± SD.

## Results

### Stability of the fish oils

There was a striking difference in the peroxide values of the unstable and the Pufanox-stabilized oils; the unstable oil started at 2 mEq/kg and reached a peroxide value of 23 mEq/kg after 1 week and 47 mEq/kg after 2 weeks. No further measurement was made on this oil. The two stabilized oils started both at 1 mEq/kg, and after 1 week both oils had reached 2 mEq/kg, and after 2 weeks the DHA-rich oil had reached 3 mEq/kg, whereas the EPA-rich oil was still at 2 mEq/kg. After 9 weeks the DHA-rich oil had reached 29 and the EPA-rich oil 14 mEq/kg.

### Food intake, weight gain, plasma lipids, α-tocopherol concentration, and lipid peroxidation

Food intake and weight gain did not differ among animals fed the different diets (data not shown). Plasma triacylglycerol and total cholesterol concentrations were significantly lower in the groups fed fish oils than in the control group (Table II). Plasma α-tocopherol concentrations were higher in rats fed the two stabilized fish oils compared to control (Table II). The plasma MDA concentration was significantly higher after feeding the unstable DHA-rich oil diet compared to the other diets (Table II).

**Table II.** Plasma triacylglycerols, plasma total cholesterol, plasma α-tocopherol and plasma MDA in rats fed the different fish oil-enriched diets. Data are expressed as mean ± SD.

	Control	Stabilized EPA-rich oil	Stabilized DHA-rich oil	Unstable DHA-rich oil
Triacylglycerols (mmol/L)	1.1 ± 0.4	0.6 ± 0.2^a^	0.5 ± 0.1^b^	0.7 ± 0.2^a^
Total cholesterol (mmol/L)	1.6 ± 0.2	1.1 ± 0.1^c^	1.2 ± 0.1^c^	1.3 ± 0.1^b,d^
α-Tocopherol (μmol/mmol total lipids)	7.8 ± 0.9	8.7 ± 0.8	8.2 ± 0.6	7.0 ± 0.7^d^
MDA (μmol/L)	0.5 ± 0.0	0.6 ± 0.0	0.6 ± 0.1	0.7 ± 0.1^c,d,e^

^a^*P* < 0.05 versus control. ^b^*P* < 0.01 versus control. ^c^*P* < 0.001 versus control. ^d^*P* < 0.05 versus stabilized EPA-rich oil. ^e^*P* < 0.01 versus stabilized DHA-rich oil.

### Plasma phospholipid fatty acid composition

Significant differences in the compositions of plasma phospholipids between rats in the control group and those fed the different fish oil diets were seen for several fatty acids (Table III). Feeding the stabilized EPA-rich oil diet resulted in the highest proportions of EPA and total n-3 PUFAs, and the lowest proportions of total n-6 fatty acids, whereas the DHA-rich oil diet resulted in the highest proportions of DHA. A difference between feeding the stabilized DHA-rich oil, compared to the unstable, was a significantly higher proportion of both DHA and total n-3 PUFAs after intake of the stabilized oil. The EPA-rich oil diet resulted in the lowest n-6:n-3 ratio which was significantly different from both DHA-rich oil diets.

**Table III.** Plasma phospholipid fatty acid composition in rats fed the different fish oil-enriched diets. Data are expressed as mean ± SD.

Fatty acid^a^	Control	Stabilized EPA-rich oil	Stabilized DHA-rich oil	Unstable DHA-rich oil
C16:0	21.1 ± 0.9	23.2 ± 1.1^c^	23.4 ± 0.6^c^	22.7 ± 1.4^b^
C18:0	19.6 ± 0.6	18.6 ± 0.7	17.9 ± 1.0^b^	18.2 ± 1.3^b^
C24:0	0.7 ± 0.1	0.5 ± 0.0^c^	0.6 ± 0.1^b^	0.7 ± 0.1^e^
Total saturated	41.8 ± 0.9	42.9 ± 0.8	42.4 ± 1.0	42.1 ± 2.1
C16:1(n-7)	0.8 ± 0.2	0.9 ± 0.2	0.6 ± 0.2	0.6 ± 0.2
C18:1(n-9)	4.7 ± 0.6	4.2 ± 0.3^b^	4.2 ± 0.2^b^	4.2 ± 0.2^b^
C18:1(n-7)	2.6 ± 0.6	2.7 ± 0.2	2.1 ± 0.3^b,f^	2.1 ± 0.1^b,f^
C24:1(n-9)	0.8 ± 0.4	1.1 ± 0.1^b^	0.8 ± 0.1^e^	0.9 ± 0.2
Total monounsaturated	8.9 ± 0.9	9.0 ± 0.5	7.8 ± 0.5^c,f^	7.9 ± 0.3^c,f^
C18:2(n-6)	24.7 ± 1.1	17.1 ± 3.7^d^	18.1 ± 1.4^d^	18.7 ± 1.6^d^
C20:3(n-6)	1.5 ± 0.3	0.7 ± 0.3^c^	1.0 ± 0.4^b^	0.5 ± 0.4^d,h^
C20:4(n-6) Arachidonic acid	12.2 ± 1.3	9.3 ± 0.8^d^	12.4 ± 0.6^g^	10.7 ± 1.2^b,e,h^
C20:5(n-3) EPA	0.7 ± 0.1	7.4 ± 0.8^d^	2.5 ± 0.4^d,g^	2.7 ± 0.5^d,g^
C22:5(n-3)	0.7 ± 0.2	1.8 ± 0.3^d^	1.0 ± 0.1^g^	0.8 ± 0.1^g^
C22:6(n-3) DHA	5.6 ± 0.4	8.1 ± 0.6^d^	12.7 ± 0.5^d,g^	11.1 ± 0.6^d,g,j^
Total (n-6) PUFAs	38.4 ± 1.0	27.1 ± 0.8^d^	31.5 ± 1.4^d,f^	29.9 ± 2.6^d^
Total (n-3) PUFAs	7.0 ± 0.4	17.3 ± 0.9^d^	16.2 ± 0.4^d,f^	14.6 ± 0.8^d,g,i^
Ratio (n-6):(n-3)	5.5 ± 0.3	1.6 ± 0.1^d^	1.9 ± 0.1^d,f^	2.0 ± 0.2^d,f^

^a^Only fatty acids >0.5 g/100 g total fatty acids are shown. ^b^*P* < 0.05 versus control. ^c^*P* < 0.01 versus control. ^d^*P* < 0.001 versus control. ^e^*P* < 0.05 versus stabilized EPA-rich oil. ^f^*P* < 0.01 versus stabilized EPA-rich oil. ^g^*P* < 0.001 versus stabilized EPA-rich oil. ^h^*P* < 0.05 versus stabilized DHA-rich oil. ^i^*P* < 0.01 versus stabilized DHA-rich oil. ^j^*P* < 0.001 versus stabilized DHA-rich oil.

### Brain phospholipid fatty acid composition

Relative to the plasma phospholipid fatty acids, only minor differences could be observed in the brain phospholipid fatty acids compared to control (Table IV). Only the stabilized DHA-rich oil diet resulted in a significant increase in DHA and total n-3 PUFAs compared to the control diet. All fish oil diets showed a significant decrease in total n-6 fatty acids and n-6:n-3 ratio compared to control.

**Table IV.** Brain phospholipid fatty acid composition in rats fed the different fish oil-enriched diets. Data are expressed as mean ± SD.

Fatty acid^a^	Control	Stabilized EPA-rich oil	Stabilized DHA-rich oil	Unstable DHA-rich oil
C16:0	24.3 ± 0.7	25.3 ± 1.7	25.6 ± 1.4	24.4 ± 2.8
C18:0	17.2 ± 0.6	17.7 ± 0.9	17.3 ± 0.6	16.8 ± 1.0
Total saturated	43.2 ± 0.9	44.7 ± 1.5	44.4 ± 1.5	44.3 ± 1.5
C16:1(n-7)	1.1 ± 0.3	1.2 ± 0.2	1.4 ± 0.1	1.2 ± 0.3
C18:1(n-9)	17.6 ± 1.3	17.0 ± 2.0	16.8 ± 0.4	18.4 ± 2.2
C18:1(n-7)	3.6 ± 0.3	3.4 ± 0.3	3.5 ± 0.3	3.6 ± 0.4
C20:1(n-9)	1.8 ± 0.4	1.4 ± 0.2^b^	1.4 ± 0.1^b^	1.5 ± 0.3
C24:1(n-9)	0.8 ± 0.2	0.5 ± 0.2^b^	0.6 ± 0.0	0.6 ± 0.1
Total monounsaturated	25.4 ± 1.8	23.5 ± 1.6	24.2 ± 0.7	25.4 ± 1.8
C18:2(n-6)	0.6 ± 0.0	0.5 ± 0.0	0.5 ± 0.0	0.5 ± 0.1
C20:4(n-6) Arachidonic acid	9.1 ± 1.0	8.3 ± 0.8	8.6 ± 0.4	8.1 ± 1.3
C22:4(n-6)	2.9 ± 0.4	2.6 ± 0.2^b^	2.5 ± 0.2^b^	2.4 ± 0.2^c^
C22:6(n-3) DHA	13.5 ± 1.0	14.0 ± 1.1	15.2 ± 0.7^c,e^	14.4 ± 0.9
Total (n-6) PUFAs	12.6 ± 1.3	11.4 ± 0.4^d^	11.6 ± 0.6^b^	11.0 ± 0.3^b^
Total (n-3) PUFAs	13.5 ± 1.0	14.0 ± 1.1	15.2 ± 0.7^c,e^	14.4 ± 0.9
Ratio (n-6):(n-3)	0.9 ± 0.1	0.8 ± 0.0^c^	0.8 ± 0.0^d^	0.8 ± 0.0^d^

^a^Only fatty acids >0.5 g/100 g total fatty acids are shown. ^b^*P* < 0.05 versus control. ^c^*P* < 0.01 versus control. ^d^*P* < 0.001 versus control. ^e^*P* < 0.05 versus stabilized EPA-rich oil.

### Brain nitric oxide synthase activity

**Figure 1.**
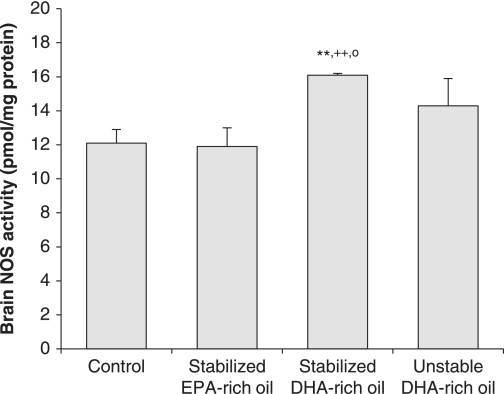
Brain nitric oxide synthase (NOS) activity (as L-citrulline, pmol/mg protein) in rats fed the different fish oil-enriched diets. Data are expressed as mean ± SD. **P < 0.01 versus control; ^++^*P* < 0.01 versus stabilized EPA-rich oil; ^O^*P* < 0.05 versus unstable DHA-rich oil (EPA = eicosapentaenoic acid; DHA = docosahexaenoic acid).

A significantly higher NOS activity in the brain was found in the group fed the stabilized DHA-rich oil diet compared to the other groups (Figure 1).

## Discussion

In this study both the EPA- and DHA-rich oil diets affected the blood lipids in a similarly positive manner (Table II), which has also been reported in studies using pure EPA and DHA oils in humans (20,21).

The lipid peroxidation of plasma (MDA) was significantly increased only after intake of the unstable fish oil (Table II). It has been shown that most commercial fish oils become rancid after only a few days owing to their deficient antioxidant stabilization (11). Also, highly concentrated and chemically modified fish oils are usually more unstable than natural fish oils. An unstable oil may consume antioxidants such as vitamin E in the body, and induce formation of free radicals leading to lipid peroxidation and cellular injury (10–13,22). The unstable fish oils can also increase blood glucose levels, probably due to lipid peroxidation in the pancreas, resulting in decreased insulin production (23). A study in humans found that the plasma lipid peroxidation induced by intake of an unstable fish oil was not inhibited by simultaneous intake of even high doses of vitamin E (24). Also in animals, vitamin E does not appear to provide sufficient protection to diets high in n-3 PUFAs (10). Although there is concern that fish oils may increase lipid peroxidation, the *in vivo* data to date are inconclusive (10,25). This might reflect that oils of different stability and in different dosages are used in the studies. Also storage and handling during the supplementation period might differ, as well as methods used to measure the oxidation.

Most of the plasma phospholipid fatty acids in this study were significantly affected by both the EPA- and DHA-rich oil diets compared to control, reflecting their specific fatty acid pattern (Table III). The unstable DHA-rich oil diet, compared to the stabilized one, resulted in less increase in the n-3 PUFAs. This could possibly reflect lipid peroxidation and is in accordance with the observed increase in plasma lipid peroxidation (MDA) in the group consuming the unstable fish oil diet. Compared to the plasma phospholipids, changes in the brain phospholipids (Table IV) were much smaller, indicating a resistance towards modification. Only the diet enriched with the DHA-rich oil, compared to the EPA-rich oil, resulted in a significantly greater incorporation of DHA as well as total n-3 PUFAs in brain phospholipids. The areas corresponding to the fatty acid EPA in the analyses were very small in the samples from brain (corresponding to <0.1 g/100g total fatty acids), and had approximately the same area in all fish oil diet groups, but were absent in the control group. By comparing the ratios of EPA:total n-3 and DHA:total n-3 of the two fish oils (Table I) it is evident that the DHA-rich oil contained relatively more of its specific fatty acid DHA than the EPA-rich oil contained EPA. This could have influenced the enrichment of EPA and DHA in the phospholipids in the two groups (EPA-rich oil diet group versus DHA-rich oil diet group) and is a drawback of not using the pure fatty acids. Synergistic effects of different fatty acids in a blend are also plausible.

The DHA content of brain is much higher than the EPA content, and DHA is a major constituent of neuronal membrane phospholipids. Since the membrane phospholipid fatty acid composition is important for the configuration and function of neurotransmitter receptors, DHA has been suggested to be the most important n-3 fatty acid for brain function (26,27). Accordingly, fatty acid preparations with high DHA content have shown a beneficial effect on brain function (26–28). For example, it has been shown that the intake of DHA, not EPA, among pregnant women is important for the intelligence of the child when tested at 4 years of age (29), and intake of DHA, but not EPA, has been shown to be associated with reduced risk of Alzheimer’s disease (30). EPA is the precursor of eicosanoids which have important functions as secondary messengers and neuromodulators (31), inhibits cyclo-oxygenase-2 (32), and has an effect on gene expression (33). Furthermore, EPA is an inhibitor of phospholipase A_2_ and may thereby decrease the release of fatty acids such as DHA from the phospholipids in the cell membrane (34). Some psychiatric disorders, such as schizophrenia, are characterized by increased activity of phospholipase A_2_ (35,36). Peet *et al*. (36) found an improvement in schizophrenic patients by EPA, but not by DHA, administration. Also, several studies have reported improvements in children with neuropsychiatric problems supplemented with an EPA-rich fish oil; however, the results have not been consistent (3,37–39).

Administration of n-3 PUFAs has been shown to improve learning ability and memory in rats and mice (40–42), and several studies have found brain NOS activity to be of crucial importance to this cognitive ability (4–9). It has been shown in piglets that supplementation with DHA significantly enhances NOS activity in the brain (43). This study in rats points to DHA as an essential constituent of a fish oil for improvement of brain DHA content and brain NOS activity. However, studies performed by us on the relaxation of vascular tissue in rats have shown that an EPA-rich oil is the most efficient (44). Thus, depending on what is under investigation, the optimal effect may be met by fish oils of different DHA:EPA ratios. This study also found that not only the fatty acid content but also the stability, and thus the antioxidant content, of the fish oil was important to brain DHA concentration and NOS activity.
